# The Emergence of Employees’ Change Readiness for Energy-Conservation Behavior During Guided Group Discussions

**DOI:** 10.3389/fpsyg.2021.587529

**Published:** 2021-11-01

**Authors:** Amelie Verena Güntner, Paul Constantin Endrejat, Simone Kauffeld

**Affiliations:** ^1^Department of Psychology, University of Zurich, Zurich, Switzerland; ^2^Department of Industrial and Organizational Psychology, University of Hamburg, Hamburg, Germany; ^3^Department of Industrial/Organizational and Social Psychology, Technische Universität Braunschweig, Braunschweig, Germany

**Keywords:** energy-conservation behavior, change readiness, resistance to change, Motivational Interviewing, guided group discussion, employee green behavior, interaction analysis

## Abstract

Studies of energy conservation efforts to reduce CO_2_ emissions in the residential sector are abundant; however similar efforts in organizations have not received as much attention as they deserve. In this study, we focus on methods for increasing employees’ readiness to change their behaviors in favor of energy conservation, specifically examining the use of guided group discussions (GGDs). We use observational research methods to examine the micro-level of behavioral dynamics and understand the emergence of change readiness. We describe how facilitators (“change agents”) can conduct GGDs and foster employees’ change readiness using the established communication approach of Motivational Interviewing (MI). We also explore how employees can increase each other’s change readiness regarding energy conservation behavior. Based on our sample of eight videotaped GGDs (5430 behavioral events), interaction analysis reveals that solution-focused communication elicits change readiness in employees, whereas problem-focused communication prompts resistance to change. We further show that employees can motivate their co-workers to express “green” intentions: when employees verbalized statements in favor of energy saving, this increased other employees’ change readiness, while verbalized statements against energy saving had the opposite effect. This demonstrates that GGD participants are active individuals who can spark behavior change in their co-workers. Finally, based on our findings we propose several communication guidelines for working with groups and discuss the importance of solution-focused energy management practices to facilitate change readiness for energy saving in the workplace.

## Introduction

Human behavior has a significant impact on climate change (e.g., Dietz et al., 2007; Wynes and Nicholas, 2017). To mitigate climate-related challenges to the natural environment, actions need to be taken that go beyond pro-environmental behavior in private households (Lo et al., 2014; Leygue et al., 2017). Today, organizations account for 50–60% of energy use (Stern et al., 2016). Thus, reducing energy emissions within organizations is mandatory if we are to meet the climate goals formulated in the 2015 Paris Agreement. While the construction of energy-efficient buildings is one way to address energy conservation, finding ways to decrease users’ consumption of energy may offer more opportunities for energy saving than those available through architectural and technical strategies alone (Janda, 2011). However, organizational members (e.g., employees) do not operate in a vacuum; rather, their attitudes, intentions, and behavior towards energy conservation in the workplace are affected by their co-workers (Paillé et al., 2016). This means that energy management practices that require active participation from employees should take into account social influence, for example through gathering groups of employees to collectively find ways to conserve energy. In this paper, we show how these participatory interventions could be designed. We also zoom in to examine the micro-dynamics of behaviors that happen during one of these interventions, a guided group discussion (GGD).

Compared to individuals in the residential context, individuals in the workplace perceive fewer opportunities and feel less responsibility for energy conservation (Murtagh et al., 2013). Furthermore, performing green behaviors in the workplace can be perceived by employees as too time-consuming, unfeasible to implement, or as having little impact, generating a tension between organizational and environmental goals (Carrico and Riemer, 2011; Bull and Janda, 2018; Hengst et al., 2020). To overcome these barriers, employees need to build “change readiness,” defined as the “beliefs, attitudes and intentions of change target members regarding the need for and capability of implementing organizational change” (Armenakis and Fredenberger, 1997, p. 144). To encourage change readiness, participatory approaches such as GGDs that involve those expected to change have been considered promising in the pro-environmental literature (Norton et al., 2015; Endrejat and Kauffeld, 2018). Furthermore, organizations frequently rely on dedicated individuals, often labeled “change agents,” who are responsible for communicating and promoting the desired change (Benn et al., 2015). For these change agents, the key question is: How can they successfully steer a given participatory setting toward their desired outcome? Such group interventions can often take on a dysfunctional dynamic, as participants may take the opportunity to complain and express their resistance to the change (Kauffeld and Lehmann-Willenbrock, 2012). In this study, we draw on self-determination theory (SDT; Deci and Ryan, 1985; Ryan and Deci, 2000) as a theoretical framework to help answer this question, as in previous research examining pro-environmental behavior change (e.g., Pelletier, 2002; Webb et al., 2013; Endrejat and Kauffeld, 2018). The fundamental assumption of SDT is that individuals naturally strive toward growth and self-actualization, and that individuals are guided by the fulfillment of three basic psychological needs, namely autonomy, relatedness, and competence (Deci and Ryan, 1985). The theoretical underpinnings of SDT are considered in the practical method we introduce, Motivational Interviewing (MI; Markland et al., 2005; Miller and Rollnick, 2013). MI has been shown to be effective in various settings related to behavior change (e.g., addictive behavior change and organizational change; Grimolizzi-Jensen, 2017; Magill et al., 2018). MI emphasizes the need for solution-focused communication that respects individuals’ autonomy and evokes their own readiness for change (Miller and Rollnick, 2013). In our study, we demonstrate how change agents (i.e., energy managers) trained in MI can successfully build employees’ readiness for positive behavior change (energy conservation at work) by means of autonomy-supportive and solution-focused communication.

MI has been successfully applied to increase motivation for green behaviors in both dyadic (Klonek et al., 2015) and group settings (Endrejat et al., 2020). However, we still lack a sound understanding of the temporal dynamics surrounding the emergence of employees’ change readiness, as well as of how employees can encourage change readiness for energy conservation behaviors in their co-workers. That is, given the workplace context, change agents need to consider the fact that employees’ attitudes and behaviors are not isolated from other workgroup members affected by the change. Rather, change readiness is influenced by the behavioral dynamics within a group of employees during a GGD. Thus, in addition to the influence a change agent may have on employees, employees may also influence each other’s reactions to change. Building on the basic psychological need for relatedness, as described in SDT (Deci and Ryan, 1985), we argue that employees may have two potential effects on each other’s change readiness: they may positively reinforce change readiness, or they may trigger downward motivational spirals (Kauffeld and Meyers, 2009; Endrejat et al., 2020).

The goal of the present research is to contribute to the literature on the facilitation of energy conservation behaviors in the workplace in the following ways. First, we describe how GGDs, as participatory interventions, help to build employees’ readiness for behavior change. Second, we show how MI helps change agents to steer this process. Third, we apply observational research methods that allow for the interaction analysis of change agents’ and employees’ behaviors, thus overcoming the shortcomings of self-report studies (see Lange and Dewitte, 2019). Fourth, we use this observational approach to highlight how employees themselves can prompt both readiness for and resistance to change in each other. In sum, the current study seeks to open the black box of successful change communication, specifically by contributing to a better understanding of how a positive change in organization members’ energy conservation behaviors can be facilitated in a group setting.

### Using GGDs to Motivate Employees Toward Behavior Change

Research on employee green behavior typically distinguishes between behaviors that constitute a mandatory part of an employee’s job and behaviors that employees execute voluntarily (Norton et al., 2015). Most commonly, employees are not forced to engage in energy conservation behaviors. They therefore need to perform extra-role energy conservation behaviors at their own discretion. For the purposes of the present research, we define energy conservation behaviors as behaviors that involve employees’ personal initiative, including turning off equipment or lights and using temperature control systems (Norton et al., 2017). In other words, for successful energy management practices to take place, organizations are highly dependent on the support of the employees, who are expected to change their behaviors for the better (Boiral, 2009). Thus, there is the need to direct employees toward change-supportive attitudes and behaviors, thus promoting increased change readiness (Jimmieson et al., 2009).

When an organization’s goal is to reduce its energy costs, employees might support the idea of looking for ways to reduce their energy consumption at the office. However, they may also express ambivalence, being concerned that such energy conservation behaviors will occupy too much time during work hours (Piderit, 2000). In the work context, one can assume that most energy conservation activities are not a source of inherent joy. However, individuals do appear to engage in them out of some sort of inner commitment as a result of extrinsic motivation. As stated by SDT (Deci and Ryan, 1985; Ryan and Deci, 2000), extrinsic motivation differs depending on the Perceived Locus of Causality (PLOC; DeCharms, 1968). In the case of an external PLOC, an individual feels that their actions are controlled by external forces. An internal PLOC indicates a deeper assimilation, in which an individual feels that they have autonomy in their actions (Gagné and Deci, 2005). The process during which individuals become autonomously motivated is called internalization, meaning that “external regulation of a behavior is transformed into an internal regulation and thus no longer requires the presence of an external contingency” (Gagné and Deci, 2005, p. 334). For example, if employees support energy conservation measures not only to avoid conflicts with their supervisors but also because they value the means and ends of the measures, the likelihood of successful change increases. Importantly, the internalization process does not happen automatically (Vansteenkiste and Sheldon, 2006). Instead, organizations need to promote contexts and interpersonal interactions in which this process can take place (Dumitru et al., 2016). One way to foster this process and eventually increase change readiness is to take into account change recipients’ psychological needs, such as autonomy and relatedness, by means of participatory interventions (Gagné and Deci, 2005; Endrejat and Kauffeld, 2017).

We combine these assumptions regarding employees’ internalization processes with the perspective that employee readiness for behavior change is more than an individual factor located within employees. Instead, we build our research on the view that reactions to change are socially constructed, emerging from the discourses circulating at a given time (Mumby, 2005; Putnam et al., 2005). To facilitate these discourses, we conduct GGDs, which involve employees in the decision-making process. In this paper, the GGDs we refer to are structured workshop discussions led by a change agent. These discussions give employees the opportunity to reflect on which energy conservation behaviors are applicable in their workplace and how these can be incorporated into daily work routines. GGDs are guided, following the idea that simply letting people talk is not enough; their interactions should be facilitated in direction of attitude and behavior change (Werner and Stanley, 2011). The challenge for change agents in encouraging change readiness in employees is exacerbated by the fact that employees tend to be ambivalent about change, often experiencing conflicting attitudes, opinions, or goals. In most cases, there are some factors promoting a target behavior (driving forces) and others that prevent individuals from showing a desired behavior (restraining forces) (Lewin, 1947a). For change to occur, the driving forces must be strengthened, the resisting forces weakened, or a combination of the two. Previous research indicates that individuals may express perceived driving and restraining forces through the language they use; that is, their language may express either change readiness (“change talk”) or resistance to change (“sustain talk”) (Endrejat et al., 2017). Such talk in turn is thought to reflect employees’ reactions to change, and represents an important mechanism of behavior change (Ladd et al., 2018). Previous research (e.g., Magill et al., 2018) has suggested that higher proportions of change talk are related to desirable outcomes, such as behavior change. In the present study, we use GGDs as a way of bringing groups of employees together so they can discuss their opinions and behaviors around energy conservation at work and agree on measures to reduce energy consumption. As employees are given the opportunity to discuss their views and share their ideas and concerns, those employees who are initially ambivalent may feel supported to embrace the requested behavior change (Werner et al., 2008). That is, the use of GGDs is based on the importance of hearing others’ views in fostering behavior change, as this provides individuals with useful behavioral information (Lewin, 1947b; Prislin and Wood, 2005). In the present study, we investigate the concrete behavioral dynamics taking place between change agents and employees over the course of various GGDs. In doing so, we aim to identify *how* and *why* employees’ verbalized reactions to change (i.e., change readiness and resistance to change) vary dynamically through social interactions.

### Using MI to Facilitate Change Readiness for Energy Conservation Behavior

Given that it is unlikely that change agents will prompt change readiness through lecturing (Lewin, 1947b), we first examine the degree to which employees’ readiness to change derives from social interactions between change agents and employees. The communication dynamics between change agents and employees are said to influence fluctuations in employees’ change readiness (Elving, 2005). In this regard, it is important that change agents use their communication to prompt employees to talk themselves and each other into supporting the change (Miller and Rose, 2009).

In previous research on organizational change management and employee green behavior, insufficient or ineffective communication has emerged as a dominant theme in explaining resistance to behavior change (for a review, see e.g., Yuriev et al., 2018). One study found that poor organizational communication about energy saving, for example if employees feel “ill-informed about the concrete benefits of relevant behaviors and what behaviors they could perform themselves” (Lo et al., 2012, p. 234), negatively affects employees’ feelings of self-efficacy (perceptions of the behaviors that one can engage in), thus undermining change readiness (Keller et al., 2019). Instead, successful communication skills shown by change agents that respect employee autonomy can promote change (Bordia et al., 2004; Li et al., 2020). A closer look at micro-level communication behaviors, revealing how change agents interact with employees about energy conservation behavior, is a promising avenue toward the provision of guidelines for such conversations.

Even today, change agents can find it challenging to communicate behavior change in a way that avoids attempting to persuade employees to change their behaviors, an approach that is likely to prompt resistance (Senge, 2010). Such “autonomy-restrictive communication” (Klonek et al., 2015) includes confrontations or warnings (“Think about the negative consequences for the environment”), preaching (“You shouldn’t use your colleagues’ behavior as an excuse”), and advising without being asked (“I would suggest you start printing fewer documents”). This communication style usually prompts employees to argue against the change (Klonek et al., 2014; Zanin and Bisel, 2018). The reason behind this response is that when individuals perceive that their freedom is threatened they might choose to argue, not against the suggested change in particular but for the sake of restating their (perceived) autonomy (Brehm, 1980). We know from previous research that individuals with autonomous motivation are more likely to show voluntary green behavior (Norton et al., 2015). Autonomous motivation, as described in SDT (Deci and Ryan, 1985; Ryan and Deci, 2000), describes a motivational state in which individuals feel self-determined to choose whether they engage in a certain behavior or not. Specifically, perception of autonomy in relation to task assignments has been found to be positively associated with energy conservation behavior (Siero et al., 1989). Given the importance of this need to experience autonomy, change agents who communicate with employees should recognize that the importance of self-determination when deciding on which change measures are to be implemented and how (Webb et al., 2013; Dumitru et al., 2016; Ruepert et al., 2016; Li et al., 2020). We argue that GGDs led by a change agent who is proficient in motivational communication methods can serve as an ideal foundation to preserve employees’ feelings of autonomy while building their readiness for organizational change.

One approach that highlights the need for autonomy and the self as driver of one’s own actions is MI (Miller and Rollnick, 2013). MI is a “collaborative, person-centred form of guiding to elicit and strengthen motivation for change” (Miller and Rollnick, 2009, p. 137). MI was originally developed in a clinical setting, as a method for clinicians to talk to their clients and motivate them in relation to the treatment of substance use disorders. Beyond that, further research has considered MI a promising approach for various settings related to behavior change, including coaching (Passmore, 2007; Klonek et al., 2016), organization development (Grimolizzi-Jensen, 2017; Güntner et al., 2019), and also specifically pro-environmental behavior (Forsberg et al., 2014; Endrejat et al., 2015; Klonek et al., 2015). MI is founded on the idea that instead of lecturing people, it is more productive to emphasize autonomy and implement change initiatives in a way that fits with employees’ preferences and the given work environment (Miller and Rollnick, 2013). In this regard, MI postulates that employees already have everything they need to achieve change, and that readiness to engage in behavior change is facilitated through communication between agents and employees (Miller and Rollnick, 2013). Nonetheless, even when employees are willing to engage in behavior that facilitates change, they might also voice reasons that weigh against a behavior change (Piderit, 2000). MI gives considerable attention to ambivalent language from individuals, emphasizing that they have a voice that speaks in favor of change (the “change talk”) and a voice that speaks against change (the “sustain talk”). By using MI, change agents can draw on a variety of communication methods that help them to listen carefully to their employees in order to identify and foster employee change talk, which is associated with higher readiness for change (Magill et al., 2018).

The efficacy of MI in evoking change readiness can be explained by the proficient use of particular verbal methods, such as using open questions and reflections to gain empathy and avoiding pressuring, lecturing, and coercion (Miller and Rose, 2009; D’Amico et al., 2015). When using these communication methods, change agents need to make sure that they formulate their questions and reflection in a way that focuses on solutions. From a solution-focused perspective, it is more effective to inquire about how a desired state (e.g., frequent engagement in energy conservation behaviors) can be achieved, rather than focusing on hindrances in a more problem-focused communication style (Watzlawick et al., 1974). Thus, an MI-based intervention aims to evoke statements from employees that signal their support and capabilities for implementing change. MI acknowledges that the language we use shapes our social realities (Fairhurst and Grant, 2010), and can function as a self-produced cue by which individuals can ascertain the change readiness of others based on their expressive behavior (Rafferty et al., 2013). Accordingly, instead of simply asking open questions, change agents should explicitly ask employees to provide some reasons that they should change their behavior (Apodaca et al., 2016). We expect that such solution-focused questions are likely to elicit “change talk” from employees (Lehmann-Willenbrock et al., 2015). As well as knowing how to encourage motivation for change using solution-focused questions, it is also important to understand how this change readiness can be maintained and reinforced. We assume that change agents who specifically reflect employees’ change-supportive statements will in turn elicit more change talk in their employees, thereby activating resources (e.g., self-efficacy) that help to embrace the change process (Cohen and Sherman, 2014). We therefore hypothesize that:

*Hypothesis 1a:* Change agent’s solution-focused communication elicits employees’ verbalized change readiness.

Conversely, change agents that rely on problem-focused communication are likely to cause employees to express resistance to change. Problem-focused communication may include statements through which change agents paraphrase employees’ negative expressions (e.g., “You say that you don’t see any benefits in saving energy at your workplace”) or questions that ask for any perceived hindrances to energy saving (e.g., “What’s preventing you from using your laptop’s energy-saving mode?”). This type of communication focuses on hindrances and encourages employees to talk about the problems they perceive in executing energy conservation behaviors at work. Hence, we predict that when a change agent encourages employees to talk about their personal reasons against change (e.g., by asking questions that inquire about problems associated with energy conservation), this will cause employees to express resistance to change. Likewise, change agents who affirm employees’ expressions of resistance may invite them to further elaborate on the reasons against change, thereby launching downward motivational spirals during which employees collectively argue that certain change measures cannot be implemented in their daily jobs (Kauffeld and Meyers, 2009; Kauffeld and Lehmann-Willenbrock, 2012). It has previously been shown that strategies like “letting off steam” and “venting” do not lead to positive outcomes in an intervention (Rosen et al., 2021) and might even make the normal problems associated with behavior change loom larger (de Shazer et al., 2007). We therefore hypothesize that:

*Hypothesis 1b:* Change agent’s problem-focused communication elicits employees’ verbalized resistance to change.

### How Motivational Contagion Emerges in GGDs

To ensure sustainable behavior change, change agents must make use of employees’ own initiative to introduce changes in the workplace (Grant and Parker, 2009). Regardless of the role of change agents in eliciting readiness for green behavior change, the role of employees themselves in successful adaptation to change therefore becomes equally critical. The question of the influence of change agents on employees’ verbalized change readiness leads to a second set of questions, namely the influence of employees’ communication styles on the change readiness of their co-workers.

Behavior change does not happen in isolation; rather it takes place within the social context of a particular work environment. Thus, groups can be considered a resource through which individuals mobilize to create social transformation (Snow et al., 1986). In regard to energy conservation, the norms within an individual’s workgroup or broader professional network are thought to influence their behavior (Darley and Beniger, 1981; Cialdini and Trost, 1998). For example, if an employee is concerned that his or her co-workers will not approve of energy conservation behaviors, he or she is likely to express resistance to these behaviors. However, when an employee perceives that his or her co-workers will be well-disposed toward energy conservation behaviors, he or she is likely to respond more favorably to these behaviors (Werner and Stanley, 2011). Werner (2003) has shown that employees’ perceptions of the kinds of green behavior that other participants would endorse influences the extent to which employees approve of such behavior themselves (see also Lewin, 1947a).

Given that human action is driven by the fulfillment of basic psychological needs, we can assume that employees participating in a GGD will be motivated by the basic need of relatedness. This means that they are likely to be concerned about what other participants might think of them. Relatedness involves the need to experience connectedness with others and to have satisfactory and supportive social relationships (Deci and Ryan, 1985). Thus, in order to experience relatedness with their group members, individual employees are likely to align their verbalized reactions to change with the verbalizations of their co-workers. Specifically, we propose that the alignment processes taking place during GGDs can be considered processes of “motivational contagion” (Endrejat et al., 2020). Motivational contagion describes the process in which an expression of change readiness by one participant increases the likelihood that another participant will also voice change readiness. For example, employees who may still be indecisive about the change can be encouraged by other employees within the group who are more motivated toward change. In this regard, employees may serve as mutual support when it comes to overcoming hindrances that lie in the way of realizing change (Wagner and Ingersoll, 2013).

Research on interactional processes in organizational groups indicates that every statement made by a group member has an influence on subsequent statements from other members and on overall team effectiveness. For instance, Kauffeld and Lehmann-Willenbrock (2012) showed that functional interactions, such as proactive statements, resulted in greater participant satisfaction with the meeting. Proactive statements may, for example, indicate an interest in change or relate to the planning of concrete steps following the meeting. Employees within the group who reflect on the positive aspects of energy conservation, or who voice statements in favor of it, might act as role models and mobilize more ambivalent employees (Endrejat and Kauffeld, 2018). We therefore hypothesize that:

*Hypothesis 2a:* Expressions of employees’ change readiness elicits other employees’ verbalized change readiness.

Previous research indicates that processes of motivational contagion can also occur in a negative way, thereby undermining employees’ readiness for change. One study in the context of workgroup meetings, for example, showed the negative effects of dysfunctional communication in team meetings (Kauffeld and Lehmann-Willenbrock, 2012). This research emphasized the negative relationship between critical statements and participant satisfaction with the meeting and the success of the team. These findings are supported by other research stating that conflict-based dynamics in teams lead to the demotivation of team members (Chen et al., 2011). We want to test the possibility that employees who reflect on possible concerns about energy conservation, or who voice problems about it, increase their co-workers’ verbalized resistance to change. Put formally, we hypothesize that:

*Hypothesis 2b:* Expressions of employees’ resistance to change evokes other employees’ resistance to change.

## Materials and Methods

To test our hypotheses, we used a multi-method design based on quantitative and qualitative evaluations of observational video data. Data were gathered in the context of a research project that had the goal of reducing CO_2_ consumption at a German university by 40%. To reach this target, university departments were offered a GGD addressing the topic of energy conservation. This GGD was structured by a facilitator from the organizational psychology department who had received 14 days of formal MI training. The goal of every GGD was to identify potential energy conservation measures within departments that would apply specifically to the participants’ own workplace environments.

### Procedure and Participants

We collected data from five GGDs at a German University that were structured around the following process steps (Endrejat et al., 2017): (1) *engaging*; (2) *collecting energy-saving ideas*; (3) *force field analysis*; (4) *strategies to increase driving and reduce restraining forces*; and (5) *action plan*. First, the change agent conducted a change readiness assessment (Armenakis et al., 1993) by asking participants to indicate how motivated they were to participate in this workshop. Second, the groups brainstormed potential energy conservation possibilities. During the third step, a force field analysis (Lewin, 1947a), participants answered two questions related to each energy conservation opportunity: (A) what hinders them in engaging in this energy conservation behavior at their workplace (restraining forces), and (B) what motivates them to engage in this energy conservation behavior at their workplace (driving forces). In the fourth step, the groups discussed how the impact of the restraining forces could be reduced and how the impact of the driving forces could be enhanced, and then decided on specific strategies to be transferred into an action plan. In the final step, the development of an action plan, the groups assigned tasks and responsibilities according to the strategies that had been identified.

In total, 75 employees participated at the GGDs, which were conducted at the Institute of High Voltage Technology and Electrical Facilities (13 team members), the Institute of Psychology (21 team members), the IT service center (17 team members), the Institute of Energy and Process Systems Engineering (15 team members), and within an interdisciplinary project team (nine team members). All workshops were facilitated by the second author. Due to privacy concerns, we are unable to provide personal information regarding participants’ demographic variables. The GGDs were recorded on video after all participants had given their informed consent. The average length of the GGDs was 124 min (SD = 16.15; Min = 106; Max = 144). Participants were asked to ignore the videotaping and to act as they would under normal circumstances. Because the participants showed no visible signs of feeling observed (e.g., they appeared to be comfortable making negative remarks about their organization), we can assume that they largely ignored the camera. This mitigates concerns related to social desirability.

#### Unitizing and Coding Process

Video data from the five GGDs were unitized and then coded using the INTERACT software (Mangold, 2014). Consistent with Bales’s (1950) approach, (1) the video was cut into units (i.e., the smallest speech segment that expresses a complete thought); (2) the person speaking was identified (i.e., change agent or employee); and (3) a corresponding behavioral code was assigned. This fine-grained procedure allowed us to assign one mutually exclusive code to each sense unit and to code every single verbal expression during the GGD in an exhaustive way. Since not all codes from the coding scheme we used were relevant to our hypotheses, we did not consider all of them in our analysis. In total we analyzed 5,430 coded behaviors. Because the GGD length varied, we followed established standards in the interaction coding literature (Bakeman and Quera, 2011; VanLear, 2017) and standardized the number of behavior counts. Standardization of behavior counts is commonly used to ensure comparable results across different human interactions that vary in length. In our study, we accounted for differences in workshop length by standardizing the behavior counts per decentile to a 120-min period using computed behavior rates. For instance, if a workshop took 100 min instead of 120 min, we multiplied the codes for this sample by 1.2 (i.e., 120/100). Similarly, if a workshop took 140 min, we multiplied the codes in this sample by 0.8 (i.e., 120/150). In doing so, we ensured that results were not confounded by the length of the workshops.

The unitizing and coding procedures were conducted by two trained research assistants. To establish inter-rater reliability, we followed guidelines in observational research and randomly selected 25% of the final sample to be double coded (Bakeman et al., 2005). Cohen’s kappa value (Cohen, 1960) was 𝒦 = 0.62 for change agent codes and 𝒦 = 0.69 for employee codes, which can be classified as strong according to the cut-off criteria of Sachs (1999), in which <0.40 = poor, 0.41–0.61 = considerable, 0.61–0.80 = strong, and 0.81–1.00 = excellent agreement. For details on kappa classification, see Bakeman and Quera (2011).

Coding of the verbal utterances in the GGDs was conducted using the German version of the Motivational Interviewing Skill Code (MISC-d; Klonek and Kauffeld, 2012). This coding scheme allowed us to measure the verbal behaviors of both employees and the GGD facilitator. While this coding instrument was initially developed to assess the quality of dyadic MI conversations between change agents and single individuals, previous studies have also applied it in a group context (e.g., D’Amico et al., 2015). The MISC-d differentiates between 19 codes for change agents; however, we only used a subset of the whole coding scheme. Regarding the change agent’s verbal behavior, we were interested in the codes for open questions and reflections that were either solution-focused or problem-focused (see Table 1). Moreover, the MISC-d differentiates between 15 codes in categorizing the verbal behavior of employees. These codes can be aggregated into three categories: “change talk” (verbal behavior that expresses readiness to change), “sustain talk” (expressions of resistance to change), and “neutral talk” (verbal behavior that is unrelated to the subject of change).

**TABLE 1:** Overview of Motivational Interviewing Skill Code (MISC-d) behavior codes.

MISC-d Code	Example
Change agent	
Solution-focused communication	What would be a positive effect of saving energy? (open question) You consider sustainability to be an important topic. (reflection)
Problem-focused communication	What is hindering you from using the energy-saving mode of your laptop? You do not see any benefits in saving energy at your workplace.
Employee	
Change readiness	We as a department need to act against a high energy consumption.
Resistance to change	I do not have the time to take care of energy-saving.
Neutral	Do I have to write anything down?

*The table only depicts the MISC-d codes (Klonek and Kauffeld, 2012) relevant for the present study.*

#### Lag Sequential Analysis

To test hypotheses H1a and H2b, we used lag sequential analysis, implemented using the Generalized Sequential Querier software (Bakeman and Quera, 2011). Through this kind of statistical analysis, we can test whether the conditional rate of a particular type of verbal behavior by one speaker after a statement from another speaker is significantly higher than the expected rate. In other words, this analysis allows us to model how the verbal behavior of one speaker affects the verbal behavior of another speaker. In our study, we used this method to evaluate how the verbal behavior of the change agent affected employees’ responses, as well as to test for processes of influence amongst employees. Sequential associations between behaviors were analyzed using time lags. These analyses estimate the interdependence of the given behavior (lag0) and the first following behavior (lag1). To determine the strength of the interdependence between specific verbal behaviors, adjusted residuals (ADJRs) are calculated. ADJRs describe standardized raw residuals (z-values) based on the difference between observed and expected frequencies (Bakeman and Quera, 2011). ADJRs greater than 1.96 indicate a significant positive association (*p* < 0.05). Based on these statistics, we can determine whether a sequential association between a given verbal behavior at lag0 and a target behavior at lag1 is significantly more or less likely than expected by chance (Bakeman and Quera, 2011).

## Results

### Descriptive Analyses

Table 2 shows the descriptive statistics for each of the behaviors by the change agent and employees of interest for the present study. Of the open questions and reflections offered by the change agent, 55.35% were solution-focused, 13.37% were problem-focused, and 31.28% were coded as neither solution- nor problem-focused (i.e., neutral). To provide evidence for the MI proficiency of the change agent who facilitated the GGDs in the present study, we computed the frequencies and percentages of the different behaviors used by the change agent. The majority of change agent behaviors were coded as neutral (64.49%), 34.00% were coded as MI-consistent, and only 1.53% were coded as MI-inconsistent. Next, we determined the percentage of the change agent’s MI-consistent behaviors in relation to all MI-consistent and MI-inconsistent behaviors (MI-consistent/(MI-consistent + MI-inconsistent), as suggested in previous research (Miller et al., 2008). A value of 95.71% indicates MI proficiency, highlighting the skills of the change agent.

**TABLE 2:** Means, standard deviations, and intercorrelations of study variables.

Variable	*M*	SD	1	2	3	4
1. CA Solution-Focused Communication	45.24	29.40				
2. CA Problem-Focused Communication	10.31	11.45	0.16	0.62^*a*^		
3. Employee Change Readiness	192.73	82.83	0.36*	–0.18		
4. Employee Resistance to Change	78.90	51.45	0.03	0.54**	0.12	0.69^*b*^

**N* = 24. CA, Change Agent. Data refer to the frequencies of behaviors within a 2-h period to account for differing lengths of GGDs.*

*^*a*^Kappa value for change agent codes.*

*^*b*^Kappa value for employee codes.*

*^∗^*p* < 0.05.*

*^∗∗^*p* < 0.01.*

Next, we analyzed the frequencies and proportions of employee behaviors. The most frequent employee behaviors were neutral behaviors (48.47%), followed by change talk (35.58%). Only 15.95% of employee behaviors were classified as sustain talk.

### Interaction Analyses Between Change Agent and Employees

To test for interdependence between the change agent’s and the employees’ behaviors, we generated lag-sequence matrices with agent behaviors and employee behaviors in the rows (i.e., a given behavior at lag0) and agent response behaviors and employee response behaviors in the column of the matrix (i.e., target behaviors at lag1). First, we conducted lag sequential analyses with the change agent’s behavior as given behavior and employees’ behavior as target behavior. Hypothesis 1a predicted that solution-focused communication from change agents would elicit change readiness in employees. The results showed that transitions from the change agent’s solution-focused communication to employee change talk were significantly above chance (*z* = 6.56, *p* = 0.01) at Lag1. These results support hypothesis 1a. Hypothesis 1b predicted that problem-focused communication from change agents would elicit resistance to change from employees. The results of lag sequential analyses showed that when change agents engaged in problem-focused communication, this increased the likelihood that change employees would produce sustain talk (*z* = 6.27, *p* < 0.01), supporting hypothesis 1b. In the next step, we conducted lag sequential analyses with employee behavior as the given behavior and behavior by other employees as the target behavior. Hypothesis 2a predicted that employees’ expressions of change readiness would elicit verbalized change readiness from other employees. In line with hypothesis 2a, change talk by one employee increased the likelihood that another employee would voice change talk (*z* = 2.67, *p* < 0.01). Hypothesis 2b stated that when employees voiced resistance to change, this would prompt other employees to express resistance to change. Results showed that sustain talk by one employee increased the likelihood that another employee would also engage in sustain talk (*z* = 6.09, *p* < 0.01), thereby supporting hypothesis 2b.

## Discussion

The present research examined the emergence and development of employees’ readiness for energy conservation behaviors during GGDs. We identified the behavioral dynamics unfolding in change agent-employee and employee-employee interactions as factors influencing both employees’ change readiness and their resistance to change. To understand these behavioral dynamics, we drew upon SDT (Deci and Ryan, 1985) as a theoretical framework. As a communication approach that builds on this theoretical framework, we introduced MI and showed how it can help change agents to guide participants through a GGD. Our results indicated that when discussing a change, focusing on solutions rather than on problems increases employees’ change readiness. These findings support and extend the idea that the behavioral dynamics evolving between change agents and employees during a GGD influence employees’ verbalized change readiness (Burnes, 2015). More specifically, our findings support the use of the communication methods provided in MI by change agents to create change readiness in a group setting, such as a GGD, to channel employee green behavior in a desired direction (Endrejat et al., 2020). In line with our understanding of employees as individuals who actively engage in the change process, we also investigated the idea that employees expressing statements in favor of change might trigger verbalized change readiness in their co-workers, finding that this was indeed the case. We also found that employees’ verbalized resistance to change triggers more resistance to change in other employees.

### Theoretical Implications

Our findings have several theoretical implications. First, our research contributes to a better understanding of change readiness (e.g., for green behavior) as a dynamic phenomenon. Contrary to most studies that examine energy conservation behavior, we did not rely on employees’ self-reports, instead using observational research methods to show that an individual’s communication style can influence the degree to which other individuals express views in favor of change or against it. For example, whereas considerable research has shown that individuals’ energy conservation is influenced by the opinions of others, it is also evident that individuals downplay the effect of others’ opinions on their own behavior when directly asked about this (Cialdini, 1985; Nolan et al., 2008). Through using behavioral observations, we avoid the biases to which questionnaire data is prone, such as social desirability (Kormos and Gifford, 2014). Thus, our behavioral research methods approach follows recent calls to avoid relying solely on self-reports when investigating pro-environmental behavior (Lange and Dewitte, 2019).

Second, our research demonstrates how the positive impact of participatory interventions, such as GGDs, can be enhanced by using the MI communication approach. Previous research has demonstrated the effectiveness of GGDs in encouraging pro-environmental attitudes and behaviors (e.g., Werner, 2003; Werner et al., 2008, 2012). We extend this previous research by providing specific guidelines on how change agents (e.g., energy managers) should communicate in order to channel change interventions, such as GGDs, in a desirable direction that goes beyond buzzwords and fancy slogans (Zorn et al., 2000). Our findings emphasize the importance of building employee change readiness by means of autonomy-supportive communication that focuses on individuals’ own ideas and solutions regarding how to change their energy consumption at work. This study also supports previous research that shows how change agents’ autonomy-restrictive behaviors can evoke resistance to pro-environmental behavior (Klonek et al., 2015). In this regard, our paper is also in line with recent research that highlights the benefits of listening instead of telling in order to motivate employees (Van Quaquebeke and Felps, 2018). Our work also relates to previous research that has called for organizations to become autonomy-promoting contexts that encourage active engagement by their employees in green behavior change (Dumitru et al., 2016).

Third, our study extends previous research on the active role of employees in energy management practices. Our findings showed that change readiness verbalized by an employee increased the chances that another employee would voice change readiness. Furthermore, when employees verbalized resistance to change, this increased the likelihood that a co-worker would voice resistance to change. These findings suggest not only that change agents can use a particular communication style to steer a group of employees in a desired direction, but also that employees themselves may have a similarly powerful impact on their co-workers’ change readiness or resistance. We showed that behavioral linkages between employees hint at the occurrence of contagious processes within group interactions and are in line with research on contagious effects during group interactions, which were previously only found in a clinical research setting (D’Amico et al., 2015; Shorey et al., 2015). Furthermore, our findings extend previous research on GGDs that showed that statements in favor of pro-environmental behaviors were related to participants’ perceptions of whether others would endorse these behaviors (Werner et al., 2008). Specifically, our study uncovered the underlying communicative dynamics between employees, which unfold as a reaction to employees’ expressions in favor of or against energy conservation behaviors. Taken together, these findings suggest that there may be benefits to raising employees’ awareness that their own behavior is formative in terms of creating supportive group norms (Lewin, 1947a).

### Practical Implications

First, the results of our study have implications for energy managers or supervisors who are frequently assigned to the role of change agents, responsible for motivating employees to undertake pro-environmental behavior change at work. Based on our findings, we suggest that change agents consider using an MI-consistent communication style when interacting with employees. This means that change agents should not attempt to “sell” change behaviors to employees who are not even contemplating change, as this approach is likely to elicit a vast number of reasons to not undertake the change (Miller and Rollnick, 2013). Instead, change agents should be sensitive to the utterances of employees, as these signal their degree of change readiness (Prochaska et al., 2001). Employees should not be urged to contribute towards an organization’s change goal; instead, they should be encouraged in their role as experts on their own work environment who can identify potential change activities and take ownership of the change (Kykyri et al., 2010). Such an approach to elicit change readiness also fits well with a proactive inquiry-based management style (Armenakis et al., 1993; Van Quaquebeke and Felps, 2018) and is in line with the finding that controlling approaches are less successful in implementing consumer behavioral change (Webb et al., 2013). Hence, we propose a communication training program in which energy managers in their role as change agents can learn the MI mindset and methods that will help them to facilitate GGDs (see, e.g., Güntner et al., 2018).

Second, our result that employees evoked each other’s readiness and resistance to change through their communication imply that employees in a change intervention are not passive recipients: they can also influence the behavior of their co-workers (Oc and Bashshur, 2013). This means that employees participating in a GGD can help steer their workgroup toward energy conservation behaviors. Specifically, employees who already show a high level of change readiness may function as “multiplicators” or “role models” for those co-workers who are still ambivalent or even resistant to the implementation of energy conservation behaviors. To help employees step into the role of multiplier and to give them confidence in motivating their co-workers to engage in behavior change, organizations could consider offering developmental programs in which employees are made aware of the influence that their own communication style can have on their peers.

### Limitations and Further Research

We acknowledge several limitations of our study. First, while we measured employees’ verbalized readiness for change, we did not incorporate objective meter data indicative of employees’ energy saving behavior into our analyses. Hence, future research should investigate whether the level of employee change readiness following a GGD translates into less measurable energy usage. However, overcoming this limitation by measuring employees’ energy consumption at the individual level is often impossible due to privacy concerns (Bolderdijk et al., 2013). For instance, assessing the amount of computer usage by a given employee would also provide information about their working hours. Nonetheless, future research should aim to develop a research design that allows for the establishment of a causal link between employees’ change readiness and actual behavior change.

Second, due to the study’s organizational setting, we could not use a randomized controlled trial. Thus, we cannot rule out the possibility that employees who were not interested in changing their energy consumption behaviors stayed away from the GGDs. This possibility is heightened by the fact that the employees’ expressed change readiness was already relatively high at the beginning of the GGDs, and that we found no difference in this measure between the beginning and the end of the GGD. In this respect, we further acknowledge the limitation that our study took place in a context (i.e., a university) that may already have a strong propensity for pro-environmental behaviors and lifestyle in general. Because our findings may not be easily transferable to other contexts, we suggest that future studies collect data and try to replicate our findings in non-academic contexts, such as larger companies that are unrelated to the energy sector.

Third, we operationalized employees’ change readiness in terms of their verbally expressed agreement to perform a behavior related to the change. However, we did not differentiate between different forms of readiness (or “commitment”), as proposed in the three-component model of organizational commitment (Meyer and Allen, 1991). These researchers labeled the three components “affective commitment” (desire to support change), “continuance commitment” (perceived cost of not supporting change), and “normative commitment” (perceived obligation to support change). Because previous research suggests that the different forms of commitment have different effects on organization-related and employee-related outcomes (Meyer et al., 2002), we encourage future studies of how the three components of commitment relate to different communication methods as used by change agents.

## Conclusion

This study contributes to opening the black box of how employees’ change readiness for energy conservation unfolds in participatory group interventions (i.e., GGDs) and how it can be facilitated using an MI-based communication approach. Our findings suggest that rather than telling employees how to change their energy consumption, change agents should use an autonomy-supportive and solution-focused approach to foster employees’ readiness for energy conservation behavior at work. Beyond the influence of change agents’ communication in fostering employees’ readiness for a change in their energy conservation behaviors, this study also identified how employees themselves can trigger verbalized change readiness in their co-workers, thereby highlighting employees’ active role in contributing to the success of organizational energy management practices.

## Data Availability Statement

The raw data supporting the conclusions of this article will be made available by the authors, without undue reservation.

## Ethics Statement

The present study involving human participants were reviewed and approved by Universität Braunschweig Ethics Committee. The patients/participants provided their written informed consent to participate in this study.

## Author Contributions

AG was responsible for the overall research ideas and model design, analyzed the data, and led the manuscript writing. PE facilitated the workshops, collected the data, and contributed significantly to writing the manuscript. SK engaged in several rounds of critical revision of the manuscript. All authors contributed to the article and approved the submitted version.

## Conflict of Interest

The authors declare that the research was conducted in the absence of any commercial or financial relationships that could be construed as a potential conflict of interest.

## Publisher’s Note

All claims expressed in this article are solely those of the authors and do not necessarily represent those of their affiliated organizations, or those of the publisher, the editors and the reviewers. Any product that may be evaluated in this article, or claim that may be made by its manufacturer, is not guaranteed or endorsed by the publisher.
